# Pneumonia: Recent Updates on Diagnosis and Treatment

**DOI:** 10.3390/microorganisms13030522

**Published:** 2025-02-27

**Authors:** Maaz Ahsan Khan, Awais Bajwa, Syed Talal Hussain

**Affiliations:** 1 Department of Internal Medicine, University of Oklahoma, Oklahoma City, OK 73104, USA; 2 Department of Pulmonary, Critical Care & Sleep Medicine, University of Oklahoma, Oklahoma City, OK 73104, USA

**Keywords:** pneumonia, syndromic panels, antibiotic stewardship, new treatments, respiratory panel

## Abstract

Pneumonia remains a leading cause of mortality internationally, making it an intense area of study for new tools for diagnosis and treatment. In this review, we evaluate the potential of recently emerging syndromic panels in promoting rapid diagnosis and improved antibiotic stewardship. We will also examine emerging treatments, including new antibiotics in a world of worsening antimicrobial resistance, in addition to new methods of delivery and non-antibiotic paths of treatment.

## 1. Introduction

Pneumonia remains a leading cause of mortality in many parts of the world, with nearly 450 million annual cases and over 4 million deaths annually from community-acquired pneumonia (CAP) alone [1]. It is commonly seen in various health settings across the world and remains an intense area of study given its significance and the gaps in knowledge that persist. In this review, we will present the more recent updates in the diagnosis and treatment of this condition.

Pneumonia is categorized into three different entities, namely CAP, HAP (hospital acquired pneumonia), and VAP (ventilator acquired pneumonia), with specific definitions from the American Thoracic Society/Infectious Disease Society of America (ATS/IDSA) guidelines. The 2019 guidelines recommend the diagnosis of community-acquired pneumonia by infiltrate on imaging, one respiratory symptom, and another finding such as fever or leukocytosis [2]. Severe CAP is a subcategory of CAP that is defined by major abnormalities in vitals or lab values, indicating a more severe infection. HAP, as defined by the ATS/IDSA 2016 guidelines on HAP/VAP, refers to pneumonia that patients acquire more than 48 h after being admitted into a healthcare facility who are not ventilated. VAP is pneumonia that develops after a patient has been on a ventilator for more than 48 h [3].

The gold standard for the identification of the microorganisms involved in pneumonia has been with culturing, either via sputum or more invasive testing such as via bronchoalveolar lavage (BAL). Biomarkers, such as procalcitonin or CRP, can help guide de-escalation, but it remains controversial if they can aid in the diagnosis of pneumonia [4]. Syndromic panels are emerging as a newer technique of microorganism identification and are further discussed below.

Treatment differs among the entities, with HAP and VAP warranting broader treatment as outlined by the 2016 guidelines. Risk factors, such as recent antibiotic exposure, colonization with MRSA, recent hospitalization, underlying lung disease further guide antibiotic selection in patients with pneumonia. This review outlines the more recent developments in the treatment of pneumonia, as well as the future directions, all of which should be of interest to clinicians considering the growing antibiotic resistance.

## 2. Syndromic Panels

A long-standing issue has been identifying infectious organisms, especially in a timely manner that can alter the clinical course. A common diagnostic tool, sputum cultures, can take more than 48 h to produce results and may only detect pathogens in less than half of patients with clinical pneumonia [5]. More invasive testing, such as obtaining cultures via bronchoalveolar lavage, can have similar rates of detection depending on the organism [6]. Additionally, the detection of commensal pathogens can make it difficult to identify pathogenic bacteria. In many cases of pneumonia, clinicians are forced to treat pneumonia empirically.

Empirical treatment of pneumonia usually involves the use of broad-spectrum antibiotics and, in recent years, as with other infections, this has led to growing rates of antibiotic resistance with pneumonia-causing pathogens worldwide [7]. The increasing prevalence of multidrug-resistant organisms will worsen global health outcomes, including the mortality from pneumonia. Appropriate antibiotic stewardship would help mitigate this issue but is difficult to accomplish when the traditional methods of culturing have limited yield.

Multiplex PCR tests have been developed in recent years to increase the yield of samples and aid in the diagnosis of, as well as the identification of pathogens in, pneumonia. They operate by utilizing PCR to detect microbial DNA for the rapid detection of a variety of common respiratory pathogens. These laboratory tests have the potential to address several of the issues with traditional culturing methods.

### 2.1. Current FDA-Approved Tests

There are currently two FDA-approved panels that can be considered true syndromic panels, with the ability to detect a wide array of organisms, including both common and more morbid bacterial pathogens.

### 2.2. BioFire FilmArray Pneumonia Panels

The BioFire FilmArray Pneumonia Panel is the most studied and well known of the available syndromic panels. It is a panel that detects eight viruses, 18 bacteria in a semi-quantitative manner, and seven different antimicrobial resistance genes [8]. It can detect samples from sputum, endotracheal aspirate, and BAL, with results available in about an hour. Many of the studies discussed below will use the BioFire FilmArray Pneumonia Panel as the syndromic panel tested.

BioFire has reported the sensitivity and specificity of their panel as 96.3% and 97.2% on sputum, respectively, with similar accuracy on BAL [8]. A meta-analysis [9] of the BioFire FilmArray Pneumonia Plus panel revealed a sensitivity of 94% and specificity of 98%.

Unique to BioFire is the semiquantitative aspect of their panel, which works by real-time PCR to estimate relative abundance. Several studies have shown good concordance between culture quantitation and semi-quantitative testing [10,11,12].

### 2.3. Unyvero Lower Respiratory Tract

The Unyvero Lower Respiratory Tract panel tests for 19 bacteria, 10 antibiotic resistance genes, and, uniquely, *Pneumocystic jirovecii* [13]. It can be used to analyze sputum cultures as well as BAL samples. It is slower than BioFire, with results produced in about 5 h. One study reported a sensitivity of 95.1% and 98.3% [14]. Please see Table 1 for a list of organisms tested by the available respiratory panels.

### 2.4. Comparison to Traditional Culturing

There have been multiple studies comparing syndromic panels to culture, and it is well documented that they have greater rates of detection as well as sensitivity, specificity, and negative predictive value [4]. Srivastava et al. [15] determined that, for the BioFire panel, the positive percent agreement with culture was 100%. The negative percent agreement varies widely in the literature, likely because growth in culture also varies widely, but has been reported as 73.2–98.1% [15]. Markussen et al. [16] demonstrated pathogen detection in 81.3% with syndromic panel testing versus 62.8% with traditional culturing. The INHALE study [17] found a detection rate of 60.4% for Unyvero, 74.2% for BioFire, and 44.2% via culture.

It is, of course, important to note that the lack of detection of organisms by these syndromic panels does not exclude pneumonia as a diagnosis, since it is based on imaging and symptoms. Syndromic panels are unable to detect every pathogen, and they will likely never be accurate enough to rule out pneumonia.

### 2.5. Evaluation of Benefits

#### 2.5.1. Rapid Identification of Pathogens

Syndromic panels can identify pathogens much more quickly than sputum cultures. The available tests can have results within hours, allowing providers to rapidly determine the appropriate antibiotics available for the pathogens and even if there are any genes for antibiotic resistance. While there has been some recent questioning about the need for quick administration of antibiotics in CAP [18], quicker administration of pathogen directed antibiotics would lead to better outcomes regarding mortality and length of stay in more severe presentations and patients with morbidities [19,20].

Several studies have shown that there is a significant improvement in the time to identification and administration of pathogen-directed treatment. The SARIPOC trial [21] demonstrated a significant difference in time to identification between critically ill patients with pneumonia tested by syndromic panels versus culturing (1.7 h versus 66.7 h). Pathogens were identified in a larger sample portion (71% versus 51%). Time to pathogen-directed therapy was 2.3 h versus 46.1 h. The CAPNOR trial [22] also recently demonstrated significance in the difference in time to pathogen-directed therapy in patients admitted for CAP, with patients receiving pathogen-directed therapy 34.5 h after syndromic panel testing versus 43.1 h in culturing. A Danish RCT studying patients presenting for CAP to the ED [23] demonstrated similar results.

#### 2.5.2. Antimicrobial Stewardship

Proponents of syndromic panels often argue that they can aid in antibiotic stewardship by prompting de-escalation more quickly, similar to how negative MRSA swabs allow for the de-escalation of anti-MRSA treatment. The IDSA/ATS guidelines recommend de-escalating antibiotics as quickly as possible once a pathogen has been identified [2]. As discussed above, traditional culturing methods make it difficult to quickly de-escalate, and in many cases when no pathogen is identified, de-escalation is not an option. As the world grapples with growing antimicrobial resistance and with a continued dearth of new antibiotics, antimicrobial stewardship remains an important practice to slow down resistance.

The INHALE trial [17,24] was a multicenter RCT utilizing syndromic panels in patients with HAP/VAP and demonstrated that, at 24 h, patients tested via syndromic panels were more likely to be receiving pathogen-directed therapy than those tested through standard culturing. The SARIPOC trial [21] showed that antibiotic de-escalation occurred in 42% of syndromic panel patients versus only 8% in culture patients, and time to de-escalation was 4.8 h versus 46.5 h, respectively.

However, other trials have shown more mixed results. A trial in Spain [25] whose primary endpoint was days of antibiotic therapy showed a modest but not significant difference between the patients admitted for CAP who had been tested via syndromic panels or culture. They reported no significant change in antibiotics despite the increased rapidity and ability of identification of pathogens. The Danish study [23] mentioned above also found, as their primary outcome, no significant difference in the prescription of no or narrow-spectrum (defined as antibiotics active against common CAP pathogens) antibiotics between the intervention and control groups. The CAPNOR trial actually showed a greater broadening of the antibiotic spectrum in patients tested with syndromic panels, in 14.3% vs. 3.9% of patients.

These mixed results on antibiotic de-escalation can potentially be explained by several factors. One, patients presenting with common pathogens for CAP are likely already on narrow spectrum antibiotics, in which case no change in antibiotics would result. This would explain why the SARIPOC trial, which evaluated critically ill patients who were likely started on broad spectrum antibiotics, showed a significant de-escalation, compared to the other trials evaluated that were studying non-critical patients.

Second, the syndromic panels have the potential drawback of detecting commensal organisms, which can make it difficult to identify the true pathogen. The BioFire FilmArray Pneumonia Plus panel tries to obviate this issue with its semi-quantitative feature, which appears to demonstrate promising use, but it has not been evaluated in an RCT specifically. Use of syndromic panels, therefore, can actually worsen stewardship by prompting the use of broader spectrum antibiotics, as observed in the CAPNOR trial.

As the discussion reveals, trials have consistently shown that patients receive pathogen-directed therapy in a more timely manner, but “pathogen-directed” is not necessarily equivalent to appropriate stewardship. It remains difficult to evaluate given the conflicting evidence, but syndromic panels may be more appropriate for severe presentations of pneumonia to promote de-escalation. Further trials are underway, namely MULTI-CAP [26], FLAGSHIP II [27], and the final results from INHALE, that should hopefully shed more light.

### 2.6. Earlier Implementation of Isolation Precautions

Syndromic panels could potentially allow for the quick identification of antimicrobial resistance, such as methicillin resistance or extended-spectrum beta-lactamase (ESBL). It has been common practice in most healthcare facilities that contact/isolation precautions are taken with these patients to prevent the spread of antimicrobial resistant strains of pathogens [28]. By rapidly identifying the patients that are either colonized or infected by these strains, these patients can be isolated quickly after entering a health facility, potentially within a few hours.

However, there have been no studies at this point studying this potential benefit, so it remains to be seen if the above-mentioned advantage of this approach can be realized. In many of the studies discussed above, there was little detection of resistance genes; however, many of the settings of these studies (Denmark, Norway) have very low rates of resistance. Populations with higher rates of resistance would likely benefit, and future research should focus on this.

### 2.7. Pneumonia—New Treatment Developments

Regarding the treatment of pneumonia in the outpatient setting for most young, otherwise healthy patients, the ATS–IDSA guidelines recommend one of the following three oral medication options for treatment of pneumonia: amoxicillin, doxycycline, or a macrolide [2]. On the other hand, those who have taken antibiotics within the past 3 months, have serious coexisting conditions, or who are smokers, amoxicillin–clavulanate and either a macrolide (preferred) or doxycycline are recommended. Patients who cannot take beta-lactam agents either due to hypersensitivity or adverse effects can instead be treated with a respiratory fluoroquinolone [2]. In addition, some risk factors such as immunocompromisation or concern for post-obstructive pneumonia, may necessitate longer treatment durations. Patients with chronic lung disease should also be considered for anti-pseudomonal coverage [29].

The choice of the appropriate antibiotic agent for the treatment of a patient who has been admitted to the hospital is based on the presence of risk factors for MRSA or *Pseudomonas* (or both). For patients admitted to a general ward without risk factors for MRSA or *Pseudomonas*, combination therapy with a beta-lactam plus a macrolide or doxycycline or monotherapy with a fluoroquinolone is recommended, though it should be noted fluoroquinolones can have increased adverse effects, particularly in the elderly [2]. If risk factors for MRSA, *Pseudomonas*, or other Gram-negative pathogens not covered by the standard community-acquired pneumonia regimens outlined above are present, coverage should be expanded. Patients with severe community-acquired pneumonia who are admitted to the ICU are more likely to be at risk for resistant pathogens, including MRSA and pseudomonas [30].

### 2.8. Recent Antibiotic Developments in Treatment of Pneumonia

With the alarming global rise in multidrug-resistant Gram-negative Bacilli, antibiotic therapy for treating patients with pneumonia can be challenging and must be guided by in vitro susceptibility results [31]. In the past few years, new antibiotics with activity predominantly against Gram-negative pathogens have been approved which, unlike older antibiotics, do not have significant side effects [32,33]. Several of the prominent antibiotics that have been approved for complicated pneumonias are listed in Table 2 [32,34,35,36,37,38,39]. Note that some are still under investigation for the treatment of pneumonia and may not have been approved by the FDA but show promise.

### 2.9. Anti-Inflammatory and Immunomodulatory Treatment

The use of glucocorticoids in the treatment of other causes of community-acquired pneumonia is evolving. The idea behind steroid use in infection is that the inflammatory response to pneumonia can cause extensive damage to the lungs, for which the immunomodulatory role of steroids can be used to decrease such damage [40]. For many years, there were several RCTs studying steroid use in pneumonia, many of which showed improvements in hospital stay duration and other measurements, but mortality benefit was controversially mixed, differing among trials and meta-analyses. The recent evidence from the CAPE-COD trial showed a benefit of survival among patients with severe community-acquired pneumonia (i.e., patients who had been admitted to the ICU and had received mechanical ventilation) and patients at high risk for respiratory failure who had been treated with hydrocortisone at a dose of 200 mg daily initially, followed by a taper [41]. Many have hailed the CAPE-COD trial as definitively concluding a mortality benefit exists, though many clinicians remain skeptical, and it is likely evidence in the future may contradict these recent results. A recent meta-analysis [42] demonstrated that it appears that only hydrocortisone, not other glucocorticoids such as dexamethasone and methylprednisolone, demonstrated a reduction in mortality. Glucocorticoid therapy has been even more controversial in influenza pneumonia, as some studies have shown increased mortality with steroid use [43,44].

Adjunctive immune therapy with different agents has been tested with limited success in severe community-acquired pneumonia. Welte and colleagues, in a phase II, double-blind study of 160 SCAP patients, compared the efficacy of a novel human polyclonal antibody preparation, called Trimodulin, which contained different fractions of immunoglobulins—IgG–56%, IgM–23%, and IgA–21%—to a placebo in increasing ventilator-free days and reducing mortality [45]. Although the study did not show significant improvement in the primary end points, a subset analyses revealed Trimodulin to have significant mortality reduction in SCAP patients who had high CRP and low IgM at baseline. Adjuvant granulocyte colony-stimulating factor (G-CSF) therapy with antibiotics in severe CAP patients did not show benefit in mortality or in the course of illness resolution [46].

Of interest has been the development of anticomplement treatments that can reduce the severity of inflammation in pneumonia, with more recent interest in COVID-19 pneumonia [47]. It is still very much an active area of research, with multiple points in the complement cascade being options for intervention and numerous pathologies with a variety of interactions with the complement system. There has been some promise shown in animal models [48], and a recent phase III trial for an anticomplement agent, vilobelimab, in the treatment of ARDS in COVID-19 pneumonia did show clinical significance compared to a placebo in decreasing mortality [49]. Another agent, AMY-101, has shown promise in its phase II trial for treatment of severe COVID-19, with plans for further trials [50].

### 2.10. New Inhaled Antibiotics

In addition to the intravenous antibiotics above, there is growing interest in aerosolized antibiotics that have the benefit of directly penetrating the site of infection. This advantage can be compounded when treating a pathogen that is only susceptible to antibiotics with limited lung penetration. Another potential advantage is reducing any systemic toxicity by administering aerosolized versions [51].

There are already several inhaled antibiotics used in the clinical setting, such as aztreonam, colistin, tobramycin, and amikacin. They have thus far not demonstrated a significant difference in mortality compared to systemic antibiotics alone, though in combination, it appears there may be a reduction in mortality. They have shown significance in clinical and microbiological cure [52]. Amikacin has more recently shown promise in preventing VAP [53].

A promising agent that is still under study is the amikacin–fosfomycin inhalation system (AFIS) that was tested by Kollef et al. [54] in a placebo-controlled trial when used in combination with IV antibiotics for VAP. Patients who received AFIS demonstrated a significant decrease in bacterial presence on their tracheal aspirates, though clinical outcomes remained similar. Further studies will be needed to better assess clinical efficacy.

Another inhaled agent under study is nebulized arbekacin, which is currently used systemically in Japan but has shown potential as an aerosolized agent in the treatment of VAP. In vitro testing demonstrated its efficacy against a wide variety of multidrug-resistant bacteria [55], and in vivo testing in murine models showed superiority over inhaled amikacin in survival rate [56].

## 3. Future Directions

Antimicrobial peptides (AMPs) have emerged as another potential therapeutic option. AMPs are endogenous peptides that are produced by the host immune system that attack invading pathogens, and there have been thousands identified in a variety of organisms [57]. They have the promise of treating MDR infections with fewer side effects and have also been found to have positive immunomodulatory effects [58]. There have been several AMP agents developed with success for the treatment of pneumonia in animal models; however, issues remain in developing an effective delivery system given AMPs’ instability in vivo due to cleavage by pulmonary proteases.

Nucleic acid agents have similar potential to AMPs. There are several RNA interference (RNAi) agents that show positive immunomodulatory effects via the transcription pathways of the immune system [58]. Further investigations are required as it has only been studied in animal models so far, though there was a recent ex vivo trial for COVID-19-infected human lung tissue with an agent that showed promise [59].

Bacteriophages have been under study as well, though their initial use actually extends back decades [58]. Phages are viruses that infect and kill bacteria, and they are found naturally in a variety of environments. They offer a number of different advantages, including targeting specific bacterial pathogens, limited adverse effects, being unaffected by antibiotic resistance, and the ability to actively seek out pathogens [60]. There have been limited studies in humans, mostly limited to individual cases, with some promising results. They are potentially limited by the time and labor consumption it can take to develop pathogen-specific phages, though this can be mitigated by phage cocktails consisting of multiple types of phages. The biggest limitation at this time is the lack of clinical trials, but growing interest will hopefully change that.

Specialized pro-resolving mediators (SPMs) represent a novel class of anti-inflammatory treatment. They are derived from fatty acids and serve in regulatory mechanisms that limit inflammatory pathways while also promoting host defense [61]. A few studies in murine [62] and simian [63] models demonstrate promise in boosting the levels of SPMs to combat pneumonia, though much remains to be studied.

## Figures and Tables

**Table 1:** The two FDA-approved syndromic panels available for pneumonia, as well as the microbes and resistance markers they detect [8,13].

	Viruses	Bacteria	Fungus	Resistance Markers
BioFire FilmArray Pneumonia Panels	Adenovirus Coronavirus Human metapneumovirus Human rhinovirus/enterovirus Influenza A Influenza B Parainfluenza Respiratory syncytial virus	*Acinetobacter calcoaceticus-baumannii* complex *Chlamydia pneumoniae* *Enterobacter cloacae* complex *Escherichia coli* *Haemophilus influenzae* *Klebsiella aerogenes* *Klebsiella oxytoca* *Klebsiella pneumoniae* group *Legionella pneumophila* *Moraxella catarrhalis* *Mycoplasma pneumoniae* *Proteus* spp. *Pseudomonas aeruginosa* *Serratia marcescens* *Staphylococcus aureus* *Streptococcus agalactiae* *Streptococcus pneumoniae* *Streptococcus pyogenes*		Carbapenemases IMP KPC NDM OXA-48 VIM ESBL CTX-M MRSA mecA/C and MREJ
Unyvero Lower Respiratory Tract		*Acinetobacter* spp. *Chlamydia pneumoniae* *Citrobacter freundii* *Enterobacter cloacae* complex *Escherichia coli* *Haemophilus influenzae* *Klebsiella oxytoca* *Klebsiella pneumoniae* *Klebsiella variicola* *Legionella pneumophila* *Moraxella catarrhalis* *Morganella morganii* *Mycoplasma pneumoniae* *Proteus* spp. *Pseudomonas aeruginosa* *Serratia marcescens* *Staphylococcus aureus* *Stenotrophomonas maltophilia* *Streptococcus pneumoniae*	*Pneumocystis jirovecii*	Carbapenemases KPC NDM OXA-23 OXA-24 OXA-48 OXA-58 VIM 3rd gen cephalosporins CTX-M MRSA mecA Penicillin TEM

**Table 2:** New antimicrobials for pneumonia treatment.

Drug	Antibiotic Class	Antimicrobial Activity
Ceftolozane + tazobactam	Cephalosporin + Beta lactamase inhibitor	Gram (−), including carbapenem and piperacillin resistant Pseudomonas aeruginosa, ESBL-producing strains
Ceftazidime + avibactam	Cephalosporin + beta lactamase inhibitor	Gram (−), including MDR P. Aeruginosa, ESBL producing strains
Ceftobiprole	Fifth generation cephalosporin	ESBL-, non-carbapenemases producing P. Aeruginosa and enterbacterales
Meropenem + Vaborbactam	Carbapenem + beta lactamase inhibitor	Gram (−) MDR organism including carbapenem resistant enterobacterales
Imipenem + relebactam	Carbapenem + beta lactamase inhibitor	Gram (−) MDR organism including carbapenem resistant enterobacterales
Cefiderocol	Cephalosporin	EBSL- producing enterobacterales; non MDR P.aeruginosa
Omadacycline	Aminomethylcycline	MRSA, Gram (−) MDR organisms
Solithromycin	Macrolide	*S. pneumoniae*, *H. influenzae*, atypical organisms
Telavancin	Glycopeptide	VRE, MRSA
Sulbactam-durlobactam	Beta-lactamase inhibitor	CRAB
Lefamulin	Pleuromutilin	CAP
Delafloxacin	Fluoroquinolone	CAP
Eravacycline	Tetracycline	CRAB
Plazomicin	Aminoglycoside	MDR gram (−) bacteria

EBSL: extended-spectrum beta-lactamases; MDR: multidrug resistant. MRSA: methicillin-resistant *Staphylococcus aureus*. VRE: vancomycin-resistant enterococcus. CRAB: carbapenem-resistant *A. baumannii.*
